# Improved Environmental Genomes via Integration of Metagenomic and Single-Cell Assemblies

**DOI:** 10.3389/fmicb.2016.00143

**Published:** 2016-02-11

**Authors:** Daniel R. Mende, Frank O. Aylward, John M. Eppley, Torben N. Nielsen, Edward F. DeLong

**Affiliations:** Daniel K. Inouye Center for Microbial Oceanography Research and Education, University of Hawai’i at Manoa, HonoluluHI, USA

**Keywords:** metagenomics, single-cell genomics, SAGs, genome assembly, microbial oceanography

## Abstract

Assembling complete or near complete genomes from complex microbial communities remains a significant challenge in metagenomic studies. Recent developments in single cell amplified genomes (SAGs) have enabled the sequencing of individual draft genomes representative of uncultivated microbial populations. SAGs suffer from incomplete and uneven coverage due to artifacts that arise from multiple displacement amplification techniques. Conversely, metagenomic sequence data does not suffer from the same biases as SAGs, and significant improvements have been realized in the recovery of draft genomes from metagenomes. Nevertheless, the inherent genomic complexity of many microbial communities often obfuscates facile generation of population genome assemblies from metagenomic data. Here we describe a new method for metagenomic-guided SAG assembly that leverages the advantages of both methods and significantly improves the completeness of initial SAGs assemblies. We demonstrate that SAG assemblies of two cosmopolitan marine lineages–Marine Group 1 Thaumarchaeota and SAR324 clade bacterioplankton–were substantially improved using this approach. Moreover, the improved assemblies strengthened biological inferences. For example, the improved SAR324 clade genome assembly revealed the presence of many genes in phenylalanine catabolism and flagellar assembly that were absent in the original SAG.

## Introduction

An enormous amount of microbial biodiversity on Earth is comprised of lineages that cannot be cultivated using traditional techniques and have been largely inaccessible to laboratory analysis (Staley and Konopka, 1985; Pace, 1997; Hugenholtz et al., 1998). Despite their influence on global biogeochemical cycles and their ubiquity in diverse environments such as soils, the ocean, and metazoan hosts, much of the physiological and phylogenetic diversity of microbial “dark matter” remains unexplored (Rinke et al., 2013; Sharon and Banfield, 2013; Hedlund et al., 2014). Over the last few decades interest in uncultivated microbial diversity has steadily increased, and new methods have been developed for its discovery and characterization (Pace et al., 1986; Lasken, 2012; Stepanauskas, 2012; Blainey, 2013; Sharon and Banfield, 2013). Chief among these methodologies are metagenomics and single-cell genomics, which both enable for draft genomes of uncultivated phyla to be sequenced and their physiological potential to be analyzed without the need for extensive laboratory manipulation (Schmidt et al., 1991; Béjà et al., 2000; Stepanauskas, 2012; Sharon and Banfield, 2013).

Both metagenomics and single-cell genomics have independently led to major breakthroughs in our understanding of uncultivated microbial diversity, but both methods suffer from distinct limitations. Because deep coverage of highly related genotypes is generally necessary for genome assembly, metagenomics to date has been most successful in recovering genes and in some cases draft microbial genomes from abundant populations (Sharon and Banfield, 2013). Moreover, assembling genomes from many microbial communities and environments remains challenging due to high genomic richness and evenness that result in a lack of high genome coverage within any specific genotype (Hedlund et al., 2014). As an alternative, single-cell genomics can be leveraged to produce partial genome assemblies from microbial cells collected from complex environments, but assembling near-complete genomes is complicated by highly biased genome coverage resulting from multiple displacement amplification (MDA; Hedlund et al., 2014). The draft genomes that result from these two different approaches are also qualitatively distinct; genomes resulting from metagenomic analyses represent “composite genomes” that incorporate genetic information from genotypically heterogeneous populations, while single cell amplified genomes (SAGs) represent genomes of individual cells that may or may not contain the full genetic repertoire present in their larger sympatric populations.

The distinct challenges and common goals of metagenomics and single-cell genomics make these technologies synergistic (Lasken, 2012; Hedlund et al., 2014), and several studies have already leveraged both methods. One study analyzing ammonia oxidation in San Francisco Bay generated five single-cell genomes and one metagenome from an enrichment culture and combined all of these sequencing datasets to yield a draft genome of the low-salinity ammonia-oxidizing archaea ‘*Candidatus Nitrosoarchaeum limnia* SFB1’ (Blainey et al., 2011). Other studies have leveraged both methods for comparative genomics; two recent studies have compared SAGs and metagenome-derived composite genomes from the candidate phylum “Atribacteria” to analyze its fermentative metabolism (Dodsworth et al., 2013; Nobu et al., 2015a), while another analyzed several SAGs and composite metagenomic assemblies of the ubiquitous SAR86 clade of marine bacterioplankton (Dupont et al., 2011). In all of these cases comparison of the draft genomes generated with different techniques allowed for novel biological insights to be drawn. Other studies have also used single-cell genomes as scaffolds for comparison or recruitment of metagenomic data when appropriate reference genomes would otherwise be unavailable, allowing for more robust analysis of metagenomic data (Eloe et al., 2011; Hess et al., 2011; Swan et al., 2013; Roux et al., 2014; Nobu et al., 2015b).

Given the overlapping goals of metagenomics and single-cell genomics, we anticipate that studies using both methodologies will become more common in the future. To facilitate the integration of these methods we developed a workflow for the combination of single-cell genomic and metagenomic data that can be used to assemble improved draft genomes from environmental samples. We present a systematic and generalized methodology that leverages this integrated approach to improve SAG genome assemblies and discuss the potential of this technique for future investigations.

## Materials and Methods

### Data Acquisition

Metagenomic sequencing data was generated from a sample taken on November 29th at a depth of 500 m from Station ALOHA on cruise #237 of the Hawaii Ocean Time-series (HOT). Metadata for this cruise is available on the website for the Hawaii Ocean Time-series Data Organization and Graphical System (HOT-DOGS) at http://hahana.soest.hawaii.edu/hot/hot-dogs/.

The two liters of water collected were pre-filtered with a 1.6 μm 42.5 mm Whatman GFA filter (Cat. No. 1820-042, Whatman) and filtrate was collected on 0.22 μm sterivex GV filter for DNA (Cat No: SVGV01015, Millipore). Cells were lysed with sucrose lysis buffer [40 mM EDTA, 50 mM Tris (pH8.3), 0.75 M Sucrose] containing 2 mg/ml of lysozyme incubated at 37°C for 30 min. Final concentrations of 1% SDS and 0.75 mg/ml Proteinase K was added and solution was incubated for 2 h at 55°C. DNA purification was performed using the FujiFilm Quick Gene instrument with the QuickGene DNA Tissue Kit (Cat. No DT-L Life Science). Libraries were created using the Illumina TruSeq LT Nano kit set A (PN: FC-121-4001). Sequencing data was generated using an Illumina MiSeq system, producing 43,359,550 individual 300 bp reads.

In this study we analyzed three SAGs generated from a clade SAR324 bacterioplankton (SAR324 cluster bacterium SCGC AAA240-J09), a clade SAR11 bacterioplankton (alpha proteobacterium SCGC AAA240-E13), and a MGI Thaumarchaeota (Thaumarchaeota archaeon SCGC AAA007-O23). The SAR324 and SAR11 SAGs were both recovered from samples taken at Station ALOHA at a depth of 770 m (Swan et al., 2011; Thrash et al., 2014), while the MGI Thaumarchaeota was recovered from a sample taken in the South Atlantic (Swan et al., 2014). We obtained the raw Illumina sequencing data for these SAGs from the DOE-JGI Genome Portal website (http://genome.jgi.doe.gov/), while the published assemblies for these SAGs were obtained from NCBI GenBank (Benson et al., 2013).

### Metagenome and SAG Assembly

**FIGURE 1:**
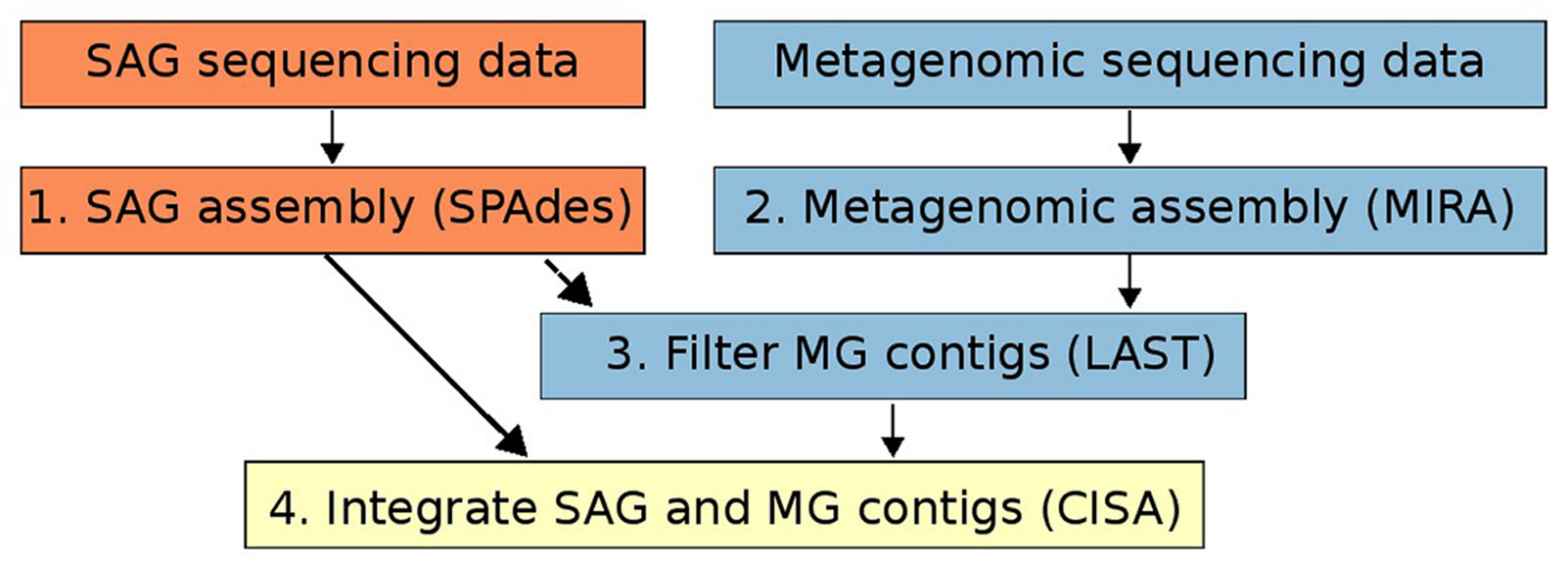
**Workflow of the method for integrating single-cell genomics (SAG) and metagenomic assemblies described in this study.** Orange boxes refer to steps involving only SAG data, blue boxes refer to steps involving only metagenomic data, and the yellow box refers to the integration of the two data types.

For both SAG and metagenomic raw data we quality filtered all raw reads using MIRA (version: 4.9.5_2) with the qc and pec options and standard parameters to retain a “high confidence region” (HCR) of every read. This step also includes the removal of contamination by phiX (Chevreux, 2004). Quality filtered SAG sequencing data was assembled using the SPAdes genome assembly program (version v.3.5.0) with default parameters (**Figure 1**, Step 1). SPAdes was chosen as an assembler as it is specifically designed for handling SAG sequencing data (Bankevich et al., 2012). For quality-trimmed metagenomic data we used MIRA (version: 4.9.5_2) to assemble metagenomic data using the standard workflow for accurate *de novo* genome assembly (Chevreux, 2004; **Figure 1**, Step 2). For both SAG and metagenomic assemblies only contigs longer than 1kbp were retained for downstream processing. Although we suggest SPAdes and Mira for SAG and metagenome assembly, respectively, in principle the assembly integration workflow presented here can be used with assemblies generated from any program (also see **Figure 1**).

### Integration of SAG and Metagenomic Assemblies

Metagenomic contigs were aligned to the newly assembled SAG contigs using LAST (Kielbasa et al., 2011) and were extracted if they matched at >95% nucleotide identity over more than 200 bp (**Figure 1**, Step 3). These contigs are putatively from the same organism or population as the SAG contigs (Konstantinidis and Tiedje, 2005). Next, we used CISA (Lin and Liao, 2013) to combine the extracted metagenomic and the SAG contigs into an integrated assembly (**Figure 1**, Step 4). For each newly generated contig, CISA reports which assembly provided the backbone. In a final filtering step, only contigs whose backbone was based on the SAG assembly were kept for the final improved SAG (iSAG) assembly. We used checkM (Parks et al., 2015) to assess both the completeness and contamination of the original and improved SAGs. Moreover, we also used the ProDeGe tool (Tennessen et al., 2015) to assess the degree of contamination both in the original SAGs analyzed and the finalized iSAGs. The results are summarized in **Table 1**.

**Table 1:** Comparison of assembly statistics of the original single-cell genomics (SAGs) and iSAGs presented here for SAR324 and MGI Thaumarchaeota.

	SAR324		MGI Thaumarchaeota	
	Original SAG	iSAG	Original SAG	iSAG
Completeness	43.67	65.78	96.88	96.88
Genome size (bp)	2,264,488	2,379,063	1,104,470	1,093,884
#Contigs	672	13	32	4
N50 (contigs)	22,317	191,983	79,020	313,273
Longest contig (bp)	94,006	354,247	217,386	319,413
GC	41.49	42.59	35.66	35.61
GC std (contigs > 1 kbp)	3.98	0.76	2.1	0.75
Coding density	87.03	89.67	92.87	93.11
#Predicted genes	2,533	2,137	1,356	1,298
#Complete genes	1,827	2,120	1,307	1,290
#Missing marker genes	84	50	4	4
#Marker genes in single copy	104	141	138	142
#Marker genes found multiple times	3	0	4	0
Contamination (checkM) (%)	0.64	0	2.07	0
Contamination (ProDeGe): (% Contigs)	89.88	0	40.63	0

*Std, standard deviation.*

### Read Mapping

**FIGURE 2:**
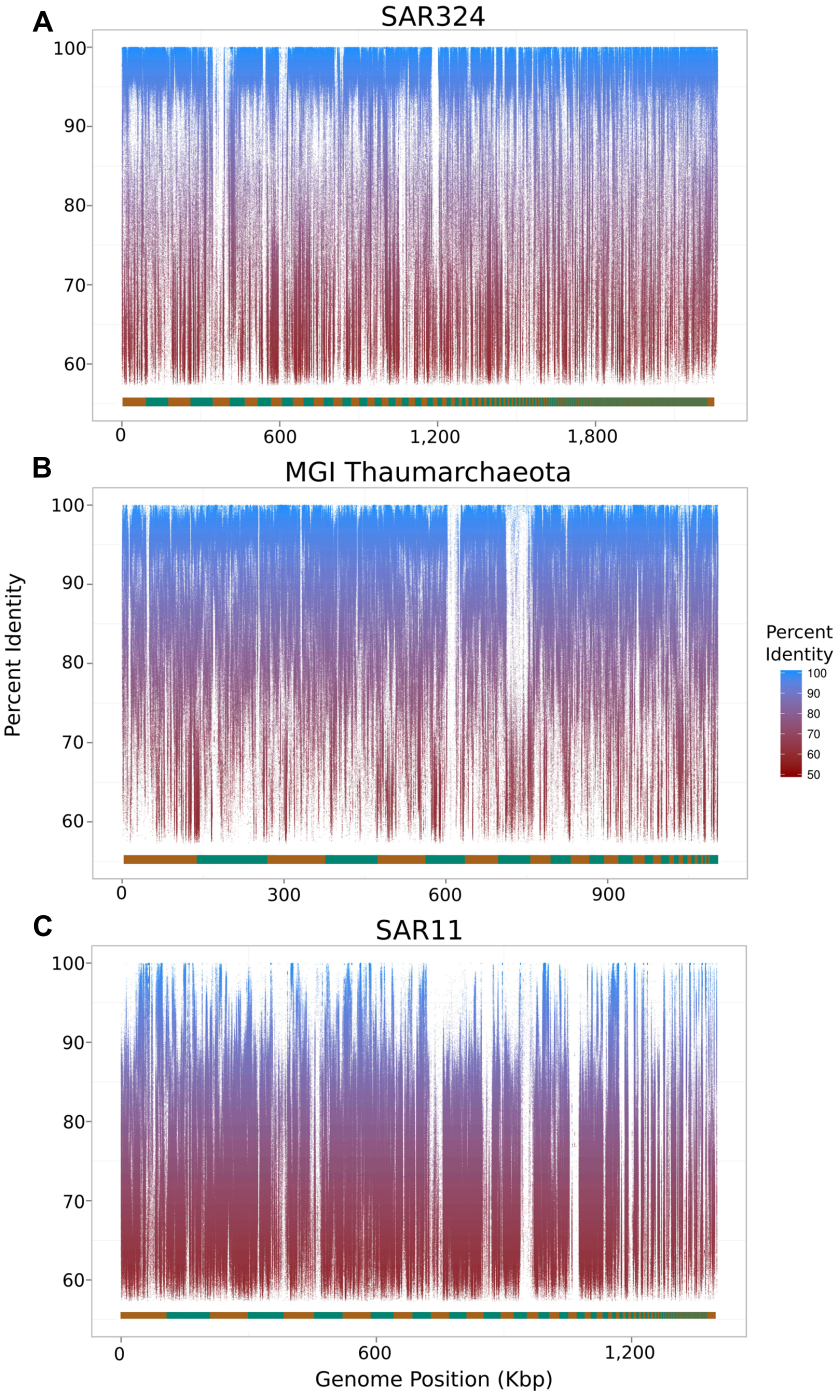
**Fragment recruitment plots of metagenomic reads mapped onto the original SAR324 clade bacterioplankton **(A)**, MGI Thaumarchaeota **(B)**, and SAR11 clade bacterioplankton **(C)** SAGs analyzed in this study.** The alternating orange and green bar at the bottom of each plot shows the contig boundaries, which are ordered from longest to shortest.

**FIGURE 3:**
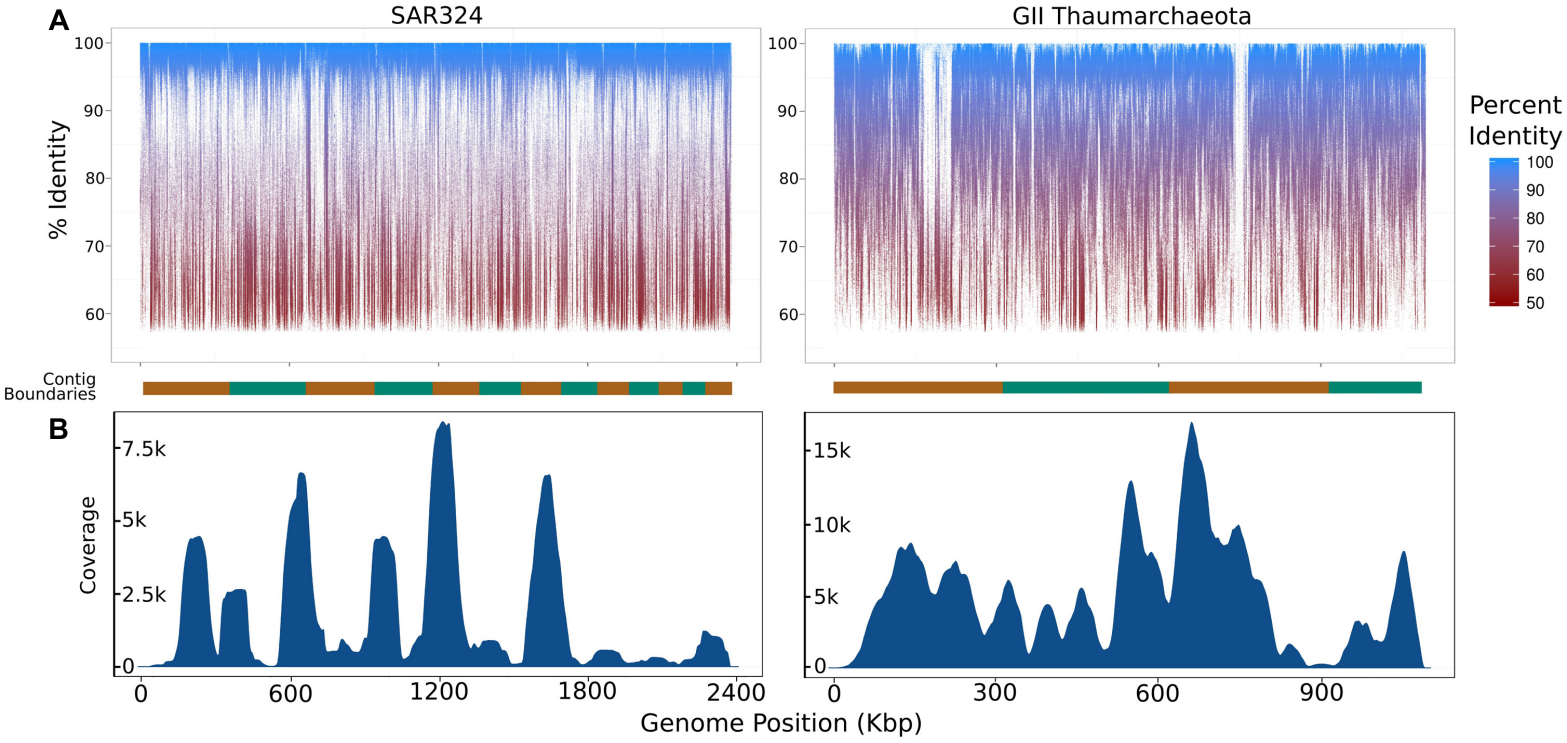
**Summary of coverage of the iSAGs assembled here in the metagenomic and single-cell datasets used. (A)** Fragment recruitment plots of metagenomic reads mapped onto the iSAGs constructed in this study. The alternating orange and green bar at the bottom of each plot shows the contig boundaries, which are ordered from longest to shortest. **(B)** Density plots showing the relative coverage of the raw SAG reads mapped onto the iSAGs constructed here.

To assess the extent to which sequences highly similar to the SAGs of interest were present in the metagenomic data we mapped metagenomic reads against the original SAG assemblies using LAST (Kielbasa et al., 2011) with default parameters. We visualized the results with fragment recruitment plots (Rusch et al., 2007) generated from the mapping data using the ggplot2 package from the R statistical programming environment (R Development Core Team, 2011; **Figure 2**). From these plots, the suitability of a metagenome for this workflow can be confirmed (as in the case of the SAR324 and MGI Thaumarchaeota SAGs) or refuted (as in the case of the SAR11 SAG; **Figure 2C**). Further, we assessed the similarity between the iSAGs and both the metagenomic sequencing reads as well as the SAG sequencing reads. For this purpose we aligned all sequencing reads using LAST and generated fragment recruitment plots (for the metagenome mapping) and density plots (for the raw SAG read mapping) to visualize these alignments (**Figure 3**).

### Functional Annotations

Annotations for the SAR324 SAG and iSAG were performed using the KEGG Automatic Annotation Server (KAAS; Moriya et al., 2007; Kanehisa et al., 2008). All complete and partial proteins predicted from these genomes were used for annotations.

## Results And Discussion

In this study we designed a workflow to combine SAGs with metagenomic data from the same environment to produce integrated, higher-quality genomes we refer to here as “improved SAGs”, or iSAGs (**Figure 1**, also see Materials and Methods). We analyzed a deeply sequenced metagenome from mesopelagic waters of Station ALOHA in the North Pacific Subtropical Gyre (presented here) together with three previously published SAGs sampled from mesopelagic waters in the Pacific and Atlantic oceans that represent the marine SAR11 clade of Alphaproteobacteria (Thrash et al., 2014), the SAR324 clade of Deltaproteobacteria (Swan et al., 2011), and MGI Thaumarchaeota (Swan et al., 2014). The success of our method varied across the three SAGs analyzed, and overall our results provide useful examples of the advantages and limitations of using metagenomic data for the improvement of assemblies generated from single-cell sequencing.

Our workflow was successful in producing iSAGs for both the SAR324 and MGI Thaumarchaeota genomes. Compared to the original SAGs, the iSAGs for these two groups were consolidated into fewer contigs, displayed increased N50 contig lengths, and contained fewer duplicate marker genes that are indicative of contamination or mis-assembly (**Table 1**). The SAR324 iSAG showed particular improvement, with an estimated 22.1% increase in completeness as estimated from conserved marker genes (65.8% from 43.7%) and an additional 115 Kb of sequence present when compared to the original SAG. The MGI Thaumarchaeota SAG was already nearly complete prior to our analysis (96%), but our workflow succeeded in consolidating the original 32 contigs into just 4, increasing the N50 contig size from 79 to 313 Kb, and reducing the estimated contamination of the contigs from 2% to 0%. Moreover, in both the SAR324 and MGI Thaumarchaeota the total number of predicted genes was reduced in the iSAG compared to the original SAG, although the total number of complete genes increased in the SAR324 iSAG (SAG: 1,827, iSAG: 2,120) and stayed relatively constant in the MGI Thaumarchaeota (SAG: 1,307, iSAG: 1,290). This reduction in fragmented genes is likely due to the consolidation of the genomes into fewer contigs, the removal of contigs <1 Kb, and the removal of redundancy and contamination, providing for more robust gene prediction and annotation.

For the SAR11 SAG analyzed, we were unable to produce an improved assembly since genotypes bearing high similarity to the original SAR11 SAG were absent, or not present in high abundance, in our metagenomic sample. Fragment recruitment plots visualizing alignments of metagenomic reads onto the three SAGs revealed that the vast majority of the SAR324 and MGI Thaumarchaeota SAGs contained high-identity matches to sequences in the metagenomic data, while this was true for only very few regions of the SAR11 SAG (**Figure 2**). This lack of representation of the SAR11 SAG in the metagenome underscores the importance of selecting SAGs that belong to populations well-represented in the cognate metagenomic data for the approach described here to be successful. Optimally, of course, the SAGs and the metagenome should be derived from the same sample population. It is possible that the SAR11 SAG analyzed here may belong to a low-abundance population that was not sampled to sufficient depth in the metagenomic data for improved assemblies to be possible. Moreover, it should be noted that in this study the metagenomes and the SAR11 and SAR324 SAGs were sampled from mesopelagic waters of Station ALOHA (770 m) at different time points, while the MGI Thaumarchaeota SAG was sampled from mesopelagic waters of the South Atlantic (800 m). Interestingly, although we were unable to improve the SAR11 SAG with our metagenomic data (both sampled at Station ALOHA in the Pacific; the former at 770 m and the latter at 500 m), we did succeed in improving the MGI Thaumarchaeota genome from the Atlantic with the same metagenomic data. We speculate that this is due to a combination of a low degree of genetic diversity in Thaumarchaeota populations, as suggested by previous studies (Hallam et al., 2006; Stieglmeier et al., 2014), in conjunction with a high abundance of this group in our metagenomic data. Despite the success of improving the MGI Thaumarchaeota SAG, however, we anticipate that the method described here would be in general most effective if used with metagenomic and SAG data sequenced from the same environment at the same time.

To further analyze the iSAGs we mapped reads from both the metagenomic data and the original SAG data back onto the new assemblies to visualize their representation in these two datasets. Similar to the mapping of metagenomic reads onto the original SAGs, mapping of this data onto the iSAGs revealed high identity matches across the majority of both genomes, with relatively few gaps (**Figure 3A**). The gaps present in the metagenome fragment recruitment plots may correspond to genomic islands that are either absent or present in low abundance in the populations sampled in the metagenomic data. This is likely to be the case for the two large gaps observed in the MGI Thaumarchaeota, especially when considering that the original SAG and metagenomic data were obtained from different water masses and likely represent distinct, albeit highly similar, population genotypes. We note that the mapping of metagenomic data back onto improved SAGs could be potentially applied to analyze population heterogeneity in a single sample. Because SAGs represent the genome of an individual cell, they may include low-abundance variants not well-represented in metagenomic data. The inclusion of both data types thus provides a more complete picture of genomic diversity in nature.

In contrast to the metagenome fragment recruitment plots, density plots showing the results of mapping SAG reads onto the iSAGs highlight the large variation in coverage typical of SAG sequencing projects (**Figure 3B**). The use of MDA in the preparation of SAGs amplifies regions inconsistently across a genome, which produces highly variable coverage in the resulting sequencing data that is a major impediment to subsequent assembly (Lasken, 2012; Hedlund et al., 2014). Our ability to improve the original SAR324 and MGI Thaumarchaeota SAG assemblies demonstrates that the inclusion of metagenomic data, which contains much less variability in coverage, allows for the joining and consolidation of many smaller contigs produced from SAG assembly alone. Use of both datasets together thus mitigates the effects of the low coverage regions biased against by MDA while making full use of the larger contigs afforded by the higher coverage regions.

**FIGURE 4:**
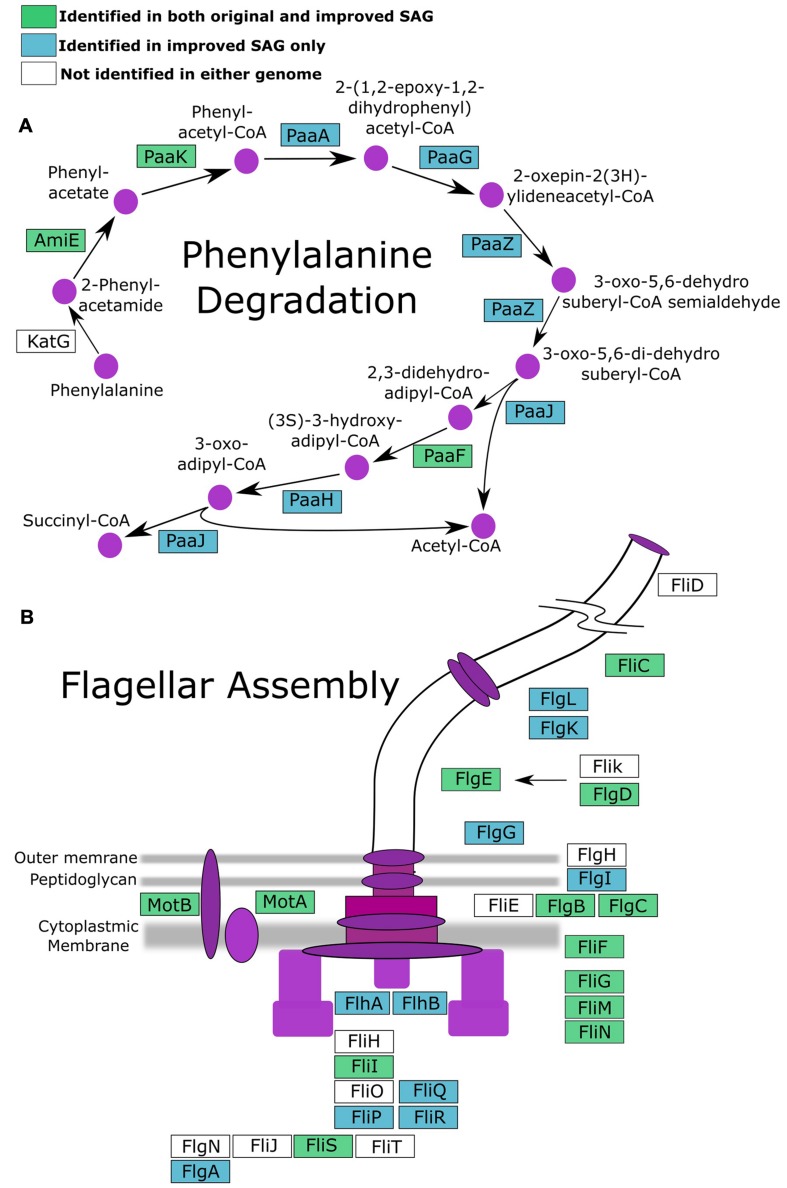
**Pathways for **(A)** phenylalanine degradation and **(B)** flagellar assembly with genes colored according to whether they were present in both the original SAG and iSAG (green), only the iSAG (blue), or neither (white)**.

To investigate if improvement of the SAG data as described here can improve biological inferences, we compared the coding potential of the original SAR324 SAG to that of the iSAG. We focused our analyses on the SAR324 genome because it showed the largest improvement in our completeness analysis (**Table 1**). Comparison of both SAR324 SAG and iSAG to the Kyoto Encyclopedia of Genes and Genomes (Kanehisa et al., 2008) revealed that many metabolic pathways were more complete in the iSAG (**Table 2**). Two of these pathways, those for Flagellar Assembly and Phenylalanine Degradation, showed particular improvement, and are displayed in **Figure 4**. For Flagellar Assembly the original SAR324 SAG encoded only 13 of the 32 genes (40.6%) in this pathway while the iSAG encoded 23 (71.9%). In the case of Phenylalanine Degradation, the original SAG encoded only genes for 3 of the 11 steps (27.3%) in this pathway, while the iSAG encoded the genes necessary for 10 of the 11 (90.9%; including two genes that encode the proteins PaaJ and PaaZ that each catalyze two reactions of the pathway). Other pathways were also more complete in the SAR324 iSAG, including those for amino acid biosynthesis, carbon metabolism, oxidative phosphorylation, and other vitamin and nucleotide biosynthetic processes (**Table 2**). Additionally, the total number of genes that could be annotated in KEGG was increased in the iSAG as compared to the SAG (SAG: 965, iSAG:1,173), consistent with our results of fewer fragmented genes in the iSAG and previous findings that consolidation of a genome into fewer contigs leads to improved gene prediction and annotation (Klassen and Currie, 2012).

**Table 2:** Comparison of KEGG Orthology protein annotations of the original SAR324 SAG and the iSAG presented in this paper.

Pathway	SAR324 SAG	SAR324 iSAG	New genes identified
Biosynthesis of amino acids	76	92	16
Phenylalanine metabolism	7	21	14
Flagellar assembly	13	23	10
Carbon metabolism	59	68	9
Two-component system	23	32	9
Oxidative phosphorylation	21	29	8
Purine metabolism	40	48	8
Cysteine and methionine metabolism	15	23	8
Ribosome	31	39	8
Glyoxylate and dicarboxylate metabolism	14	21	7
Carbon fixation pathways in prokaryotes	16	23	7
Aminoacyl-tRNA biosynthesis	16	23	7
2-Oxocarboxylic acid metabolism	12	18	6
Pyrimidine metabolism	26	32	6
Phenylalanine, tyrosine and tryptophan biosynthesis	15	21	6
Folate biosynthesis	4	10	6
Glycerophospholipid metabolism	6	11	5
Glycine, serine and threonine metabolism	24	29	5
Ubiquinone and terpenoid-quinone biosynthesis	4	9	5
Propanoate metabolism	9	13	4
Protein-coding genes with KO annotations	964	1,173	209

In this study we present a novel method for the generation of improved assemblies of environmental genomes using a combination of SAGs and metagenomic data. Results from the three SAGs analyzed in this work provide different examples of how this method performs. In the example of the SAR324 SAG we observed the largest improvement, with a significant increase in completeness, reduction in the total number of contigs, and enhanced recovery of genes and metabolic pathways. In the case of the MGI Thaumarchaeota, although the initial SAG was already near-complete, we were still able to consolidate the total number of contigs to just 4 and remove several genomic regions representing potential contamination. This was somewhat surprising given the MGI Thaumarchaeota SAG originated from the South Atlantic while the metagenomic sample originated from the North Pacific. This suggests that for population genotypes with high conservation and broad distributions the judicious application of this approach may even be used across disparate samples. Finally, the SAR11 SAG used in this study could not be improved due to a lack of abundant and highly similar genotypes in the metagenomic datasets we used, demonstrating that this method necessarily has stringent requirements for high population genotype similarity (>95% ANI) between the SAGs and metagenomes utilized. Ideally, both SAGs and metagenomes should derive from the same or similar sample, albeit for conserved population genotypes this may not be an absolute requirement. The addition of longer reads derived from newer sequencing technologies (Koren et al., 2012; McCoy et al., 2014; Goodwin et al., 2015) will likely provide additional improvement to genome assemblies in the future. Due to the widespread interest in uncultivated microbial groups in the biosphere as well as the valuable information that can be gleaned from improved genome assemblies, we anticipate that workflows such as the one described here will be a useful addition to the -omics methods currently available to researchers.

### Data Availability

Raw reads generated from metagenome sequencing are available in the NCBI Short Read Archive under accession number SRP066631. The iSAGs generated as part of this study are available in the Supplementary Information.

## Author Contributions

DM and FA designed the research, performed the analysis and wrote the manuscript. JE and TN performed the sequence assemblies and revised the manuscript critically. ED designed the research and wrote the manuscript.

## Funding

This paper is a contribution from the Center for Microbial Oceanography Research and Education (C-MORE) and the Simons Collaboration on Ocean Processes and Ecology (SCOPE). This work was supported by grants from the Gordon and Betty Moore Foundation (to ED, 3777), National Science Foundation Grant EF0424599 (to ED), and the Simons Foundation grant Simons Collaboration on Ocean Processes and Ecology (SCOPE) (to ED, 329108). DM was supported by SCOPE, EMBO (ALTF 721-2015), and the European Commission (LTFCOFUND2013, GA-2013-609409).

## Conflict of Interest Statement

The authors declare that the research was conducted in the absence of any commercial or financial relationships that could be construed as a potential conflict of interest.

## ABBREVIATIONS

MGI: Marine Group I
SAG: Single-cell Amplified Genome
